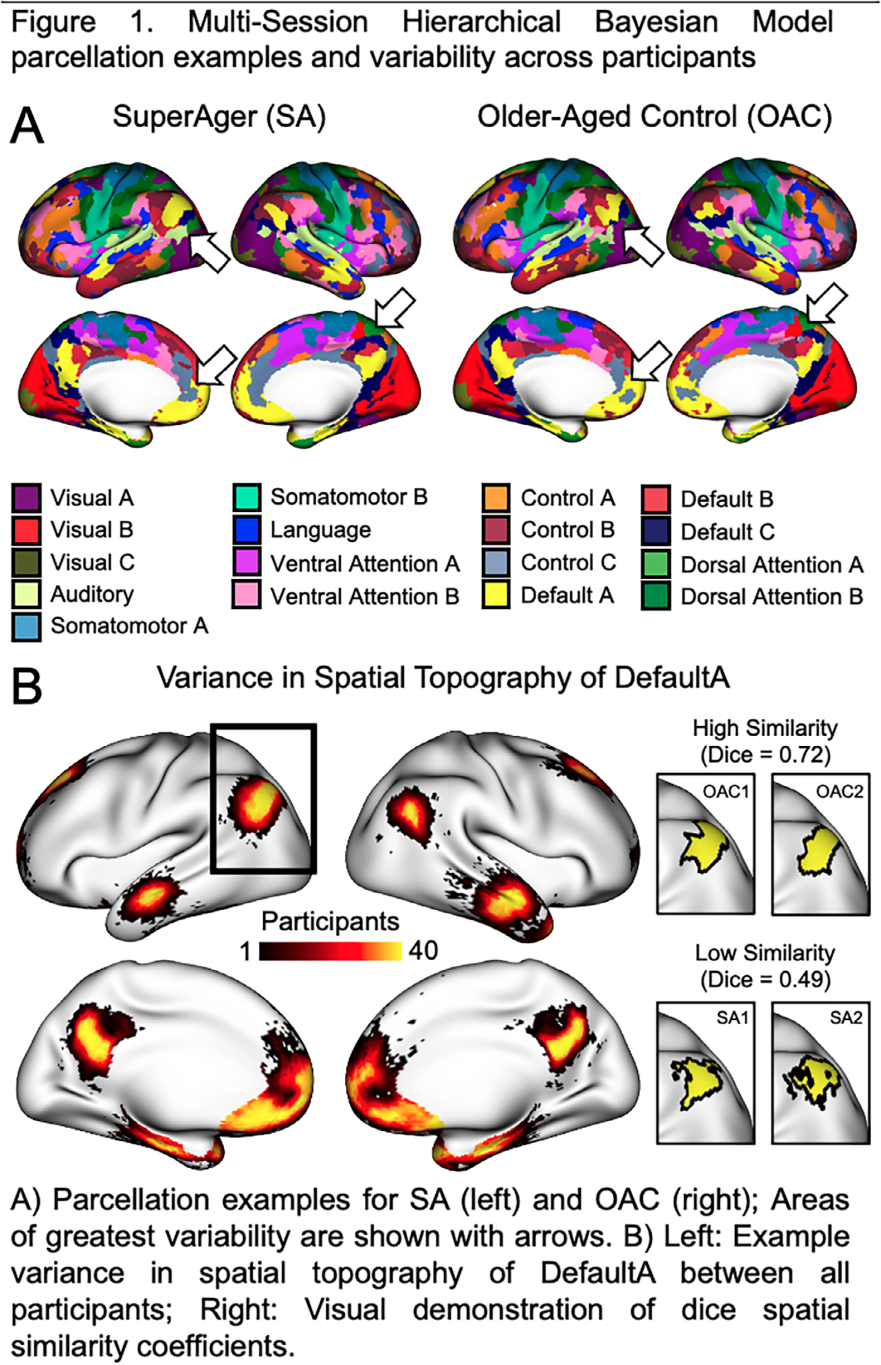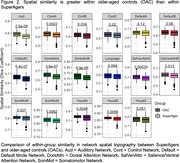# Spatial Similarity of Person‐Specific Resting State Topography in Cognitive SuperAgers

**DOI:** 10.1002/alz.090347

**Published:** 2025-01-09

**Authors:** Bram R Diamond, Adam Martersteck, Emily J Rogalski

**Affiliations:** ^1^ Mesulam Center for Cognitive Neurology, Northwestern University Feinberg School of Medicine, Chicago, IL USA; ^2^ Department of Psychiatry and Behavioral Sciences, Northwestern University Feinberg School of Medicine, Chicago, IL USA; ^3^ Healthy Aging & Alzheimer’s Research Care (HAARC) Center, The University of Chicago, Chicago, IL USA; ^4^ Healthy Aging & Alzheimer’s Research Care (HAARC) Center, Healthy Aging & Alzheimer’s Research Care (HAARC) Center, The University of Chicago, Chicago, IL USA

## Abstract

**Background:**

Memory decline in late life is a common hallmark of aging, yet SuperAgers are individuals age 80+ with episodic memory performances at least as good as cognitively average 50‐to‐60‐year‐olds. Recent work, combining anatomical and functional MRI, has shown the precise boundaries of large‐scale resting state networks vary at the individual level. Further, the use of person‐specific rather than standard parcellations has led to more behaviorally meaningful associations, and has not been explored in SuperAgers. The current project examines whether the topography of person‐specific resting state networks differ between SuperAgers and older‐aged controls (OACs).

**Methods:**

Twenty‐four SuperAgers and 16 OACs were included in the study. SuperAger/OAC phenotype was determined based on measures of episodic memory, executive functioning, verbal fluency, and picture naming across two visits, separated by at least 18 months. Person‐specific network parcellations were derived for each participant using their first visit resting state fMRI connectivity, constrained by anatomical priors, with a Multi‐Session Hierarchical Bayesian Model (MS‐HBM; Kong et al., 2021). Sørensen‐Dice spatial similarity coefficients (Dice) were calculated to determine the within‐group spatial similarity of each network. Dice was defined as the number of overlapping vertices between two participants’ network segmentation, multiplied by two, and divided by the sum of vertices in both network segmentations. Dice was calculated for each participant compared to all other within‐group participants and averaged to create a Dice score per participant per network. Dice scores for each of the 17 person‐specific networks were compared between SuperAgers and OACs using Mann‐Whitney tests, Bonferroni‐corrected.

**Results:**

Demographics did not differ significantly between groups. Person‐specific network topography varied between participants (Figure 1). SuperAgers had significantly lower Dice scores than OACs in six of 17 networks (Figure 2; p‐value ≤ 0.0029).

**Conclusion:**

Compared to OACs, SuperAgers demonstrated greater within‐group variability in resting state network topography. Greater spatial variability may reflect unique patterns of functional or structural topography that support SuperAgers’ exceptional memory abilities. Future investigations will include further examination of the functional connectome of SuperAgers, leveraging the precision approach employed here.